# Sperm microRNA Content Is Altered in a Mouse Model of Male Obesity, but the Same Suite of microRNAs Are Not Altered in Offspring’s Sperm

**DOI:** 10.1371/journal.pone.0166076

**Published:** 2016-11-04

**Authors:** Tod Fullston, E. Maria C. Ohlsson-Teague, Cristin G. Print, Lauren Y. Sandeman, Michelle Lane

**Affiliations:** 1 Discipline of Obstetrics & Gynaecology, School of Medicine, Robinson Research Institute, The University of Adelaide, Adelaide, South Australia 5005, Australia; 2 Freemason’s Foundation Centre for Men’s Health, The University of Adelaide, Adelaide, South Australia 5005, Australia; 3 Department of Molecular Medicine & Pathology and New Zealand Bioinformatics Institute, University of Auckland, Auckland 1142, New Zealand; 4 Monash IVF Group, Melbourne, Victoria 3168, Australia; INIA, SPAIN

## Abstract

The prevalence of obesity is increasing worldwide and has tripled in men of reproductive age since the 1970s. Concerningly, obesity is not only comorbid with other chronic diseases, but there is mounting evidence that it increases the non-communicable disease load in their children (*eg* mortality, obesity, autism). Animal studies have demonstrated that paternal obesity increases the risk of metabolic (*eg* glucose metabolism defects, obesity) and reproductive disorders in offspring. Epigenetic changes within sperm are clear mechanistic candidates that are associated with both changes to the father’s environment and offspring phenotype. Specifically there is emerging evidence that a father’s sperm microRNA content both responds to paternal environmental cues and alters the gene expression profile and subsequent development of the early embryo. We used a mouse model of high fat diet (HFD) induced obesity to investigate whether male obesity could modulate sperm microRNA content. We also investigated whether this alteration to a father’s sperm microRNA content lead to a similar change in the sperm of male offspring. Our investigations were initially guided by a Taqman PCR array, which indicated the differential abundance of 28 sperm borne microRNAs in HFD mice. qPCR confirmation in a much larger cohort of founder males demonstrated that 13 of these microRNAs were differentially abundant (11 up-regulated; 2 down-regulated) due to HFD feeding. Despite metabolic and reproductive phenotypes also being observed in grand-offspring fathered via the male offspring lineage, there was no evidence that any of the 13 microRNAs were also dysregulated in male offspring sperm. This was presumably due to the variation seen within both groups of offspring and suggests other mechanisms might act between offspring and grand-offspring. Thus 13 sperm borne microRNAs are modulated by a father’s HFD and the presumed transfer of this altered microRNA payload to the embryo at fertilisation potentially acts to alter the embryonic molecular makeup post-fertilisation, altering its growth trajectory, ultimately affecting adult offspring phenotype and may contribute to paternal programming.

## Introduction

Obesity and its consequences for lifelong health and well-being are an increasing burden for health systems worldwide. Globally more than 2.7 billion adults are classified as overweight or obese [1] and obesity has become the most important risk factor contributing to the overall burden of disease [2]. Adult obesity is often comorbid with many other chronic diseases, such as cardiovascular disease, type 2 diabetes, and cancer, thereby reducing life expectancy [3]. Furthermore, in men of reproductive age the prevalence of obesity has tripled since the early 1970’s; occurring with a concomitant decline in male fertility [4–6]. An increased male BMI can reduce sperm count and motility, while increasing sperm DNA damage and reactive oxygen species, and disrupting mitochondrial activity [7,8]. Consequently, male obesity is associated with increased time to conception, reduced fertilization, and impaired embryo development that culminates in reduced pregnancy rates and increased pregnancy loss [9].

Of significant concern, human epidemiological investigations provide evidence that an increased male BMI, or increased food intake, can lead to male transmission of non-communicable diseases to descendants. For example, a presumed excess of grandpaternal food was associated with reduced survivability [10] and an increased risk of diabetes [11] in grandchildren in a population from Northern Sweden (Överkalix). Furthermore, an increased paternal BMI is associated with increased BMI in his children [12–15], and paternal obesity has been associated with increased risk of autism spectrum disorder in children [16,17]. Human studies are usually confounded by a shared genetic predisposition and/or exposure to an ‘obesogenic’ environment by both the father and his children [18].

Animal models have clearly demonstrated that programming can be paternally initiated, whereby a father’s pre-conception high fat diet increases the risk of metabolic disturbances in offspring [19–21]. Using a mouse model of diet induced obesity, in the absence of overt signs of diabetes, we have previously demonstrated impaired reproductive and long-term metabolic health outcomes in offspring and grand-offspring [19,22]. Furthermore this programming of offspring health occurred concomitantly with changes to the microRNA content of the father’s sperm and germ cell hypomethylation [19].

A prime candidate mechanism, by which paternal environmental cues are passed to the next generation, are non-genetic alterations in sperm such as DNA methylation, Histone modifications, DNA damage, and in particular small non-coding RNAs (sncRNA) [23–25]. Mature sperm contain significant amounts of RNA, including microRNAs as a subset of small non-coding RNAs, which are transferred to the oocyte upon fertilization where they alter gene expression in the early embryo [26–29]. MicroRNAs are short, endogenous, single-stranded non-coding RNAs that fine tune protein expression at the post-transcriptional level by homologous binding to target mRNAs [30,31]. While microRNAs can act by translational repression [32], most act via mRNA decay [31,33]. Interestingly, microRNAs may also modulate epigenetic regulators such as DNA methyltransferases and histone deacetylases, and conversely be targets of epigenetic regulation themselves [34].

Sperm borne microRNAs have been demonstrated to be important for embryo development, as evidenced by embryonic arrest in embryos deficient of sperm derived microRNA [35] and that paternal microRNA-34c is critical for the first cleavage event [36]. Investigations that microinject supraphysiological amounts of single microRNAs into PN stage embryos provide direct evidence that alterations to microRNA abundance very early in embryo development are capable of inducing phenotypes in adult offspring. These phenotypes include cardiac hypertrophy (microRNA-1 [37]), coat colour changes (microRNA-221/222 [38]), embryo and offspring overgrowth (microRNA-124; [39]), and an obesity phenotype (microRNA-19b [40]). Furthermore microinjection of other multiple sncRNA or microRNA species are sufficient to recapitulate complex phenotypes, including offspring behavioural and metabolic defects induced by a father’s chronic stress (9 microRNAs– 29c, 30a, 30c, 32, 193-5p, 204, 375, 5323p, and 698; [41]), behavioural and metabolic defects induced by a father’s early life trauma (entire sperm sncRNA content [42]), or an offspring acquired metabolic disorder caused by a father’s over-nutrition (entire sperm tsRNA fraction [43]). The microinjection of 9 microRNAs changed in sperm by a father’s chronic stress caused targeted degradation of stored maternal mRNAs and induced a cascade of molecular events in the early embryo that ultimately induce the adult offspring phenotype [41].

We therefore hypothesised that paternal diet-induced obesity during spermatogenesis can also regulate the abundance of microRNA in spermatozoa. Therefore, our aim was to utilise our mouse model of obesity to identify sperm borne microRNAs that are altered by high-fat diet induced obesity. Furthermore given that grand-offspring born to male offspring also have metabolic and reproductive phenotypes [19] we also aimed to establish if these same microRNAs are changed in the sperm from male offspring.

## Materials & Methods

### Animals and diet

Male C57Bl6 mice (*aka* fathers; C57BL/6NHsd, Envigo, IN, USA) were fed either a control diet (CD *n* = 13 total; 6% fat, 19% protein, 64.7% carbohydrate; SF04-057, Specialty Feeds, Glen Forrest, Australia), or a high fat diet (HFD *n* = 14 total; 22% fat, 0.15% cholesterol, 19% protein, 49.5% carbohydrate; SF00-219, Specialty Feeds), which were otherwise matched for nutritional content. All male mice were housed individually post weaning prior to mating (including male offspring and grand-offspring), so a dominance structure was not established between males within the same litter that might affect sex hormones profiles and thus potentially skew reproductive and sperm microRNA outcomes. Animals had *ad libitum* access to feed and water for 10 weeks (5 to 15 weeks of age) and during experiments where fasting was not required. After 10 weeks on the diets (15 weeks of age) a subset of males (*aka* fathers; *n* = 9 per CD/HFD group) were mated with CD fed female C57Bl6 mice to generate male offspring, approximately 1 week prior to sperm collection. Male offspring (*n* = 9 per CD/HFD father; *ie* one male offspring per father/litter) were weaned and maintained on the CD until 15 weeks of age and mated with CD fed female C57Bl6 mice to generate the grand-offspring; then offspring sperm samples were collected post mortem.

The mice were obtained and housed individually by the University of Adelaide Laboratory Animal Services, Adelaide, Australia. All mice were maintained at 24°C on a 14 h light (lights on at 0600), 10 h dark (lights off at 2000) illumination cycle. All procedures were conducted during the light phase of the light cycle and all mice in each group were subjected to all experiments in the order in which they are described.

In all cohorts of mice each mouse had total body weight measured weekly and body composition determined by post mortem dissection and weighing each dissected tissue after 10 weeks of diet regimens (for fathers) or at 15 weeks of age for offspring. Tissues (including adipose tissue) and organs of each animal were dissected and weighed post-mortem.

### Animal ethics

The animal ethics committee of the University of Adelaide approved all experiments, and the animals were handled in accordance with the Australian Code of Practices for the Care and Use of Animals for Scientific Purposes.

### Metabolic testing

Following 9–10 weeks of diet treatment in fathers (14–15 week of age), intraperitoneal glucose tolerance test (IPGTT) was performed after 6 h of fasting by intra-peritoneal (ip) injection of 2 g kg^-1^ of 25% glucose solution (Sigma, St Louis, Missouri) and then one week later intraperitoneal insulin challenge (IPTT) was performed fed after ip injection of 0.75 IU kg^-1^ insulin (Actapid®, Novo Nordisk, Bagsvaerd, Denmark). Tail blood glucose concentration was obtained by a tail vein nick and measured using a glucometer (Hemocue, Angelholm, Sweden) at timepoints 0 (pre-bolus basal), 15, 30, 60 and 120 minutes. Data is expressed as mean blood glucose concentration per group using area under curve (AUC; glucose challenge) or area above the curve (AAC) analysis (insulin challenge).

### Blood hormone analysis

For blood hormone measurements, male mice were fasted overnight (14 h), and a blood sample was obtained by cardiac puncture immediately prior to sacrifice, serum was immediately separated by centrifugation (4,000 rpm for 10 m at 4°C) and frozen at -80°C until further analysis. Fasting serum glucose, cholesterol, triglycerides, non-esterified fatty acids (NEFAC test) and HDL cholesterol (HDLC3) were measured in duplicates on a COBAS INTEGRA 400 (Roche Diagnostics, Basel, Switzerland). Fasting serum insulin and leptin levels were measured in duplicates by Sensitive Rat Insulin radioimmunoassay (RIA; Catalogue # SRI 13K) and Mouse Leptin RIA, respectively (XL 85K; Millipore Corporation, Billerica, MA, USA).

### Isolation of spermatozoa

Spermatozoa were isolated from the vas deferens and cauda epididymis which had been dissected out of the mouse and gently squeezed into 1 ml GIVF medium (Vitrolife AB, Gothenburg, Sweden) with all tissue remnants removed, immediately after animal sacrifice and overnight fasting. Most of the GIVF/sperm solution was carefully aspirated, pelleted and resuspended/washed in 1 ml of PBS and counted as previously described [44] and as below, eliminating contamination of non-sperm cells. Light microscopy purity assessment confirmed samples to be entirely of spermatozoa origin. The sperm was pelleted again, snap frozen, and stored at -80°C until RNA extraction.

### Sperm count and motility

Spermatozoa were extracted into GIVF medium (Vitrolife AB) and allowed to swim out for 10 min at 37°C in 5% O_2_, 6% CO_2_ and 89% N_2_. Sperm concentration was determined using a Neubauer haemocytometer and sperm motility was determined by count of duplicate measures of 200 sperm [44] and reported as a percent of the total of motile sperm for each sample, as per the World Health Organization laboratory manual recommendations for the examination of human sperm [45].

### RNA extraction, miRNA expression profiling and bioinformatic analysis

Total RNA was isolated from 4-8x10^6^ spermatozoa for each male mouse investigated (no pooling of samples) with TRI reagent (Ambion, Waltham, MA USA), using Glycogen as a RNA carrier. RT-PCR was performed with multiplexed TaqMan primers, and the RT product pre-amplified with Megaplex PreAmp Primers. miRNA expression profiling was performed on 384-well microfluidic TaqMan Rodent MicroRNA Array cards v. 3.0 (*n* = 4 per CD/HFD group–mice from different litters were randomly allocated to the diets and then samples were randomly selected for this initial experiment) amplified on a 7900 HT Real Time PCR system. After quality control in SDS v.2.3 and RQ Manager v.1.2, the raw Ct data were quantile normalised using the normQpcrQuantile function of the R qPCRNorm package [46]. The degree of differential expression of the 371 mouse detectors that passed quality control were ranked using LIMMA in R (S1 Table). To validate the PCR array findings, qRT-PCR was performed with multiplexed TaqMan primers and the RT product pre-amplified with Megaplex PreAmp Primers, with reactions run in triplicates on a 7900 HT Real Time PCR system Data, including NTC (complete reaction without cDNA added) and no RT enzyme (complete reaction without RT enzyme) controls. miRNA expression fold change was determined by the ΔΔCT method, using the geometric mean of miR-10a and miR-195 to normalize data. Both miR-10a and miR-195 were determined to be invariable and ubiquitous endogenous controls by two approaches; cel-miR normalisation of array data (data not shown), and subsequent qPCR data (S2 Table). All reagents, machines and software were from Applied Biosystems, unless otherwise specified.

The Ingenuity Pathway Analysis® (IPA) tool was used to generate a list of mRNA targets experimentally confirmed to be direct targets of the microRNAs (*ie* strict filtering) that were differentially abundant in the sperm of HFD fathers. A core network analysis was then performed using only experimentally validated interactions between molecules (*ie* strict setting) and to predict the molecular networks that these mRNAs targets are known to function in.

### Statistics

All data are presented as mean ± SEM and checked for normal distribution by D'Agostino and Pearson test (GraphPad Prism, version 5.01 for Windows, GraphPad Software, San Diego, CA, USA) as well as equal variance by a Levene’s test (SPSS version 19, SPSS Inc., Chicago, IL, USA). Experimental outcomes were analysed using a paired Student’s t-test, a Mann Whitney U test for non-parametric data (Graphpad Prism), or univariate general linear modelling using cohort of animals as a covariate (SPSS), when appropriate. Levels of significance were set at *p* ≤ 0.05.

## Results

### A father’s high fat diet models obesity in the absence of overt signs of diabetes

We used our mouse model of high fat diet (HFD) induced obesity, where HFD consumption for 10 weeks induces obesity without any overt changes to glucose homeostasis [19,22,44,47]. Although HFD fed fathers (*n* = 14) were heavier (+24.8%), as a proportion of total body weight they had increased adiposity (+46.6%). Specifically perirenal (+65.3%), gonadal (+57.2%), and omental (+65.3%) adipose depots were greater as a proportion of total body weight (Table 1) compared with CD fed fathers (*n* = 13). Akin to human obesity, HFD feeding also increased fasted blood sera metabolite concentrations, including cholesterol (+47.3%), high density lipoprotein cholesterol low serum 3 (HDLC3; +47.5%), and non-esterified free fatty acids (FFA; +32.4%) (Table 1) compared with CD fed fathers. This HFD regimen did not alter fasted blood sera concentrations of insulin, glucose, leptin, nor responses to a glucose or insulin bolus, compared with CD fed fathers (Table 1). Thus the F0 metabolic phenotype was consistent with our previous use of this model of obesity, whereby the potential confounder of overt changes to glucose homeostasis, was avoided.

**Table 1:** Fathers total body weight, body composition, and blood metabolite profile.

Parameter	CD	HFD	*p* value
**Total body weights**	*n* = 13	*n* = 14	
Weight (g)	28.41 ± 0.75	35.46± 0.95	< 0.0001
**Adiposity–absolute mass (g)**			
Perirenal fat (g)	0.21 ± 0.04	0.42 ± 0.04	0.0010
Retro peritoneal fat (g)	0.08 ± 0.02	0.08 ± 0.03	NS
Dorsal fat (g)	0.36 ± 0.03	0.51 ± 0.04	0.0100
Gonadal fat (g)	0.88 ± 0.09	1.71 ± 0.11	< 0.0001
Omental fat (g)	0.27 ± 0.03	0.56 ± 0.04	0.0001
Sum of adipose deposits (g)	1.80 ± 0.18	3.27 ± 0.21	0.0001
**Adiposity–relative (% TBW)**			
Perirenal fat (%)	0.72 ± 0.12	1.19 ± 0.11	0.0071
Retro peritoneal fat (%)	0.28 ± 0.08	0.21 ± 0.06	NS
Dorsal fat (%)	1.27 ± 0.11	1.42 ± 0.10	NS
Gonadal fat (%)	3.06 ± 0.26	4.81 ± 0.2178	0.0001
Omental fat (%)	0.72 ± 1.19	1.19 ± 0.11	0.0003
Sum of adipose deposits (%)	6.27 ± 0.48	9.19 ± 0.41	0.0002
**Testes weights**			
Left Testis (g)	0.08± 0.00	0.08 ± 0.00	NS
Right Testis (g)	0.08 ± 0.00	0.08 ± 0.00	NS
Both Testes (g)	0.16 ± 0.00	0.16 ± 0.00	NS
**Blood metabolites**			
Glucose (mmol/L)	14.11 ± 1.80	13.69 ± 1.16	NS
Cholesterol (mmol/L)	4.40 ± 0.62	6.48 ± 0.48	0.0010
Triglyceride (mmol/L)	1.10 ± 0.14	0.98 ± 0.11	NS
FFA (mmol/L)	1.11 ± 0.16	1.47 ± 0.12	0.0045
HDLC3 (mmol/L)	3.79 ± 0.56	5.59 ± 0.43	0.0116
Insulin (ng ml^-1^)	1.71 ± 0.48	1.38 ± 0.24	NS
Leptin (ng ml^-1^)	24.87 ± 3.69	27.17 ± 2.98	NS
**Glucose/Insulin testing**			
GTT (AUC; mmol.min)	259 ± 18.6	238 ± 30.0	NS
ITT (AAC; mmol.min)	294 ± 23.7	276 ± 33.1	NS

All data is presented as Mean ± SEM.

CD Control Diet, HFD High Fat Diet, *p* value determined by 2-tailed t-test, % Weight is expressed as a percent of total body weight, NS Difference between CD/HFD is not significant (*P*>0.05), TBW total body weight, GTT glucose tolerance test, AUC area under the curve, ITT insulin tolerance test, AAC area above the curve.

### High-fat diet induced obesity affects the father’s reproductive system and spermatozoa quality

Conventional sperm analysis showed that sperm count was unaltered by HFD feeding (8.71 ± 1.01 x 10^6^/mL for CD (*n* = 13) and 8.41 ± 0.44 x 10^6^/mL for HFD (*n* = 14)) but sperm motility was significantly reduced (CD (*n* = 13)– 83.1 ± 2.6%; vs HFD (*n* = 14)– 65.1 ± 4.4%; *p* = 0.0023) compared with CD fed fathers. These data confirm our previous findings that diet induced obesity impairs sperm parameters in both mouse models and in humans [9,44].

### High-fat diet induced obesity alters a father’s sperm microRNA content

To gain a somewhat global overview of what effect diet-induced obesity has on mouse sperm microRNA content, low density TaqMan PCR arrays were used as a screening tool for 641 murine microRNAs (*n* = 4 males per CD/HFD). The expression of 371 mouse miRNAs could be detected (raw Ct<35) in all 8 CD/HFD samples. LIMMA analysis of quantile normalised Ct values of these 371 miRNAs (S1 Table) identified 28 microRNAs that were potentially differentially abundant in the sperm from HFD F0 compared with CD as determined from unadjusted *p* values (12 up-regulated and 16 down-regulated; Table 2 and full list of detected microRNAs in S1 Table). It must be noted that the microRNAs identified as potentially differentially abundant did not survive FDR testing, most likely due to the limited number of individual arrays used to interrogate them (*ie n* = 4 per CD/HFD F0 males) (S1 Table).

**Table 2:** 28 sperm microRNAs from fathers predicted to be differentially abundant in HFD F0 sperm (*n* = 4) compared with CD sperm (*n* = 4).

Array Card Assay miR ID	CD (Ct)	HFD (Ct)	HFD FC	*p* value
**Up-regulated in HFD**				
*mmu-miR-126-3p-4395339	21.87 ± 0.26	20.05 ± 0.32	3.53	0.004
*mmu-miR-135b-4395372	27.45 ± 0.31	25.77 ± 0.33	3.18	0.010
*mmu-miR-143-4395360	22.43 ± 0.26	20.29 ± 0.73	4.40	0.018
*mmu-miR-133b-4395358	27.72 ± 0.75	25.74 ± 0.29	3.97	0.029
*mmu-miR-136-4395641	28.38 ± 0.26	27.16 ± 0.31	2.33	0.030
*mmu-miR-126-5p-4373269	24.76 ± 0.49	23.32 ± 0.29	2.72	0.036
mmu-miR-376b-4395582	35.90 ± 1.65	32.42 ± 0.44	11.12	0.047
*mmu-miR-141#-002513	29.34 ± 0.29	28.35 ± 0.18	1.99	0.047
*mmu-miR-145-4395389	19.47 ± 0.49	17.82 ± 0.58	3.14	0.050
*mmu-miR-337-000193	31.98 ± 0.69	30.32 ± 0.36	3.18	0.050
*mmu-miR-30a-4373061	20.99 ± 0.46	19.81 ± 0.60	2.25	0.059
*mmu-miR-376a-4373347	31.12 ± 0.56	29.13 ± 0.60	4.14	0.059
**Down-regulated in HFD**				
mmu-miR-669n-197143_mat	27.34 ± 0.43	29.54 ± 0.37	-4.60	0.005
mmu-miR-669C-002646	28.87 ± 0.47	30.79 ± 0.28	-3.79	0.008
mmu-miR-669l-121149_mat	29.96 ± 0.51	32.14 ± 0.44	-4.53	0.010
mmu-miR-150-4373127	16.81 ± 0.33	18.27 ± 0.21	-2.77	0.011
mmu-miR-467H-002809	31.25 ± 0.65	34.65 ± 1.07	-10.56	0.015
*mmu-miR-1961-197391_mat	32.17 ± 0.51	34.97 ± 0.93	-7.00	0.018
*mmu-miR-184-4373113	19.78 ± 0.26	21.00 ± 0.32	-2.32	0.031
mmu-miR-466a-3p-002586	27.05 ± 0.44	28.37 ± 0.21	-2.49	0.033
mmu-miR-672-4395438	22.93 ± 0.22	23.87 ± 0.13	-1.93	0.036
mmu-miR-412-002575	32.94 ± 0.31	34.30 ± 1.14	-2.57	0.039
mmu-miR-669D-002808	28.82 ± 0.61	30.60 ± 0.49	-3.42	0.040
mmu-miR-139-3p-4395676	31.59 ± 0.99	35.01 ± 1.34	-10.77	0.045
mmu-miR-1969-121131_mat	29.40 ± 0.21	30.66 ± 0.20	-2.38	0.051
mmu-miR-879#-002473	32.43 ± 0.58	35.61 ± 0.54	-9.04	0.053
mmu-miR-92a-4373013	18.77 ± 0.37	19.53 ± 0.47	-1.69	0.054
mmu-miR-466b-3-3p-002500	26.72 ± 0.34	27.90 ± 0.21	-2.26	0.059

*p* value (not FDR adjusted) ranked normalised Ct values are given for the 12 up-regulated and 16 down-regulated murine microRNAs as indicated by Taqman miRNA PCR array cards. All data is presented as Mean ± SEM. Mean dCt were derived from the geometric mean of mmu-miR-10a-5p and mmu-miR-195-5p (the least variable microRNAs across all samples). A full list of microRNA detectors can be found in S1 Table.

CD Control Diet, HFD High Fat Diet, FC fold change (of HFD vs CD by ΔΔCt method), *p* value from LIMMA analysis of normalised Ct values.

* Confirmed as differentially abundant by subsequent qPCR interrogation in an extended cohort of F0 mice.

**Fig 1:**
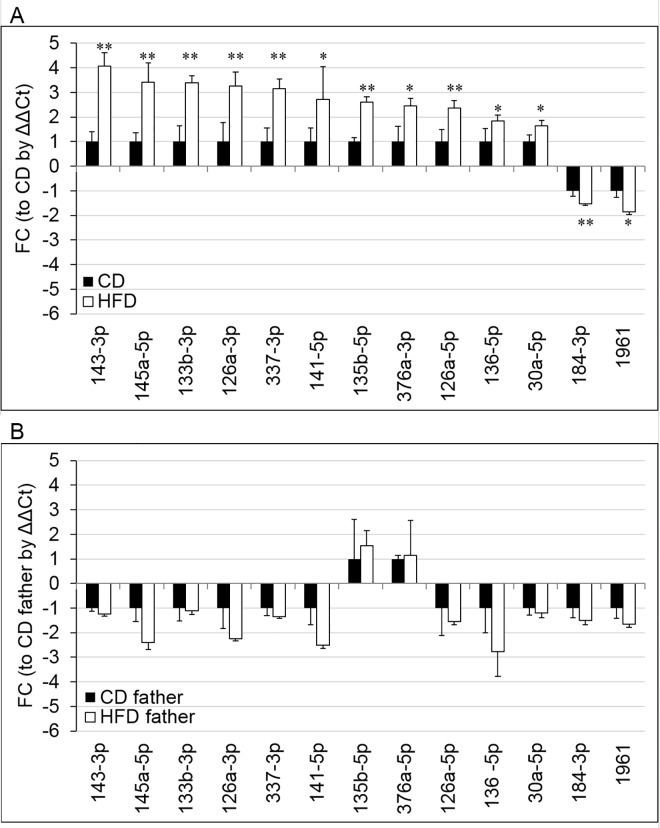
The abundance of the 13 sperm borne microRNAs by qPCR in the sperm of the (A) CD or HFD fed fathers and the (B) male offspring born to by either CD or HFD fed fathers. Fold change (FC); vs (A) CD fathers or (B) offspring born to CD fathers by ΔΔCt method) of each microRNA is given for the (A) F0 males fed the CD (*n* = 13) or HFD (*n* = 14; from 3 separate cohorts) and the (B) F1 males born to either CD (*n* = 9; black bars) or HFD (*n* = 9; white bars) fathers. The (A) geometric mean of mmu-miR-10a-5p and mmu-miR-195-5p or (B) mmu-miR-10a-5p alone was used as a reference microRNA (the least variable microRNA(s) across all samples in the present experimental setup). *p* values denoted [* *p* < 0.05; **p < 0.01] are derived from (A) univariate general linear modelling or (B) no significant differences were detected by a Student’s T test. Data is presented a means ± SEM.

To build on the preliminary array findings, individual Taqman microRNA qPCR assays for all 28 candidate microRNAs were performed on the sperm from a larger sample that included both the samples used for the array (*n* = 4 per CD/HFD F0 males) and additional males from a total of three separate cohorts of mice (*n* = 13 CD fed; *n* = 14 HFD fed). This included a subset of a cohort we had previously published that demonstrated transgenerational transmission of reproductive and metabolic phenotypes into offspring and grand-offspring due a paternal HFD [19,22]. This confirmed differential abundance of 13/28 of these miRNAs; 11 were up-regulated (mmu-miR-126-3p, 135b-3p, 143-3p, 133b-3p, 136-5p, 126-5p, 141-5p, 145a-5p, 337-3p, 30a-5p, and 376a-3p) and 2 were down-regulated (mmu-miR-184-3p, and 1961) (Fig 1, S2 Table). It must be noted that only 13/28 microRNAs were confirmed as altered in the larger cohort (Fig 1, Table 2, S2 Table), presumably due to the limited sample size of the initial cohort or variation between individual males/cohorts. Interestingly, we note a distinct difference in the proportion of microRNAs identified by the array and confirmed as differentially abundant in the up-regulated list (11/12 confirmed) compared with the down-regulated (2/16) list.

All data were normalised to the expression of two reference microRNAs (mmu-miR-10a-5p and mmu-miR-195a-5p) that we found to be invariable and ubiquitous endogenous in the Taqman PCR array data set and then validated by the individual Taqman qPCR assays in the extended cohort (S2 Table). The small non-coding RNA (U6) frequently used for normalising microRNA varied more than 10a-5p and 195a-5p across our treatment groups in our hands (S2 Table), albeit not significantly different between CD/HFD fathers. This suggests that mmu-miR-10a-5p and mmu-miR-195a-5p are potentially better endogenous controls than U6 for investigating microRNA content in mouse sperm in the present experimental setup.

In Addition 53 mouse sperm microRNAs were only detected in the sperm from either CD or HFD fed fathers (S3 Table) using a Ct of 35 as detection limit cut-off. Interestingly 13/53 were detected only in the sperm from CD fed fathers; whilst the remaining 40/53 were only detected in the sperm form HFD fed fathers.

### A father’s high-fat diet does not alter the same subset of sperm microRNAs in offspring due to variable abundance

Many paternal environmental challenges have been demonstrated to initiate transgenerational effects and epigenetic disturbances in offspring, such as endocrine disruptors [48], stress [49], pain [50], and nutrition [19,21,22,51]. We aimed to investigate whether a polygenic disease (*ie* diet induced obesity) would have the same impact on sperm microRNA content in the F1 males, who themselves have metabolic disturbances without increased adiposity. We have previously reported that the grand-offspring born through the male offspring lineage also have metabolic and reproductive disturbances (41.4 ± 10.13% decrease in serum leptin, 53.2 ± 29.99% gain in adipose tissue, insulin resistance, 33.0 ± 11.06% decrease in sperm motility, 7.2 ± 1.84% increase in sperm reactive oxygen species (ROS), and 16.2 ± 7.13% increase in oocyte ROS) that have some phenotypic overlap with those of the male offspring investigated here (6.4 ± 1.94% increase to total body weight, 22.7 ± 11.0% in serum leptin, reduced glucose clearance, insulin resistance, 17.2 ± 9.86% decrease to sperm motility, 18.0 ± 1.23% increase in sperm ROS, 38.9 ± 6.95% decrease in sperm-oocyte binding, 150.0 ± 75.12% increase in sperm DNA damage, and 80.9 ± 13.81% decrease in oocyte fertilisation) [19,22]. Thus the 13 sperm borne microRNAs dysregulated by a father’s HFD were interrogated in the sperm from the male offspring, despite their lack of exposure to a HFD and overt obesity. Consistent with another report of transmission of behavioural disturbances to offspring due to paternal stress, where the same microRNAs altered in the fathers sperm due to stress were not altered in the offspring’s sperm despite behavioural phenotypes [42], in our obese model none of microRNAs altered in the fathers showed signs of dysregulation in male offspring’s sperm (Fig 1). The lack of difference between offspring born to CD/HFD fathers was presumably due to the large variance observed in the abundance of the microRNAs in the sperm from both groups of offspring.

### Molecular pathways targeted by differentially abundant sperm microRNAs in fathers due to a HFD

An Ingenuity Pathway Analysis® with strict filtering and analysis settings was used to both (i) generate a list of 311 experimentally confirmed mRNA targets (S4 Table) of the 13 microRNAs that were differentially abundant in the sperm of HFD fathers and (ii) to predict (using a strict analysis) the molecular networks that these mRNAs operate in. The top 5 ranked molecular networks identified by this analysis, and presumably targeted post fertilisation by these sperm borne microRNAs within the embryo, included diseases and functions such as cell death and survival, cancer, cellular development, organismal development, infectious diseases/immune response, cellular growth and proliferation, and embryonic development (Table 3). P53 was identified as the highest ranked upstream regulator of these networks.

**Table 3:** The top 5 ranked molecular networks identified by Ingenuity pathway analysis.

#	Molecules in Network	IPA Score	Focus Molecules	Top Diseases and Functions
1	**Akt**, **AURKB**, **BCL2**, **BCL6**, **BECN1**, **CARHSP1**, **COL1A2**, **CSNK1D**, ERK1/2, **F11R**, **HMOX1**, **HRAS**, **IDH1**, **IGF1R**, **IGF2BP1**, **IGFBP5**, **IRS1**, **JAK2**, **KRAS**, **NRAS**, **PAFAH1B2**, **PRIM1**, **Ras**, **RB1CC1**, **RHOB**, **RTKN**, STAT5a/b, **TOM1**, **TOP2A**, **TP53**, **TYMS**, **UHRF1**, **UNG**, **VASN**, **VIM**	50	32	Cancer, Organismal Injury and Abnormalities, Reproductive System Disease
2	Ap1, **BCL2L1**, **CASP3**, **CASP9**, **CCNA2**, **CCND1**, **CDC25A**, **CDK4**, **CDK6**, **DFFA**, **EIF4E**, **FLI1**, **HMGA1**, Hsp90, **JUN**, **KLF4**, **KLF5**, **LIN28A**, **MAPK12**, **MDM2**, **MET**, **MYC**, **NFATC2**, **PKM**, **PLK1**, **PRDM1**, **PTGS2**, Rb, **RHOA**, **RUNX2**, **SRF**, **TAGLN**, **THBS1**, **TLR4**, **WNT5A**	50	32	Cellular Development, Cellular Growth and Proliferation, Cell Death and Survival
3	14-3-3, ADCY, **AKT2**, AMPK, Ap1, **ATRX**, Cg, **CTGF**, ERK, ESR1/2, **GNAI2**, **HMGA1**, **IDH1**, **KCNQ1**, **KLF15**, **KRT19**, **MET**, **MUC1**, **NEDD4**, **PARP**, Pka, PP2A, PRKAA, PTPRK, APH1A/B, **SFRP4**, **SPRED1**, **STRN**, **TGFBR1**, **UBE2I**, **UGT8**, Vegf, **VEGFA**, **VIM**, **WNT5A**	30	22	Cellular Movement, Cancer, Endocrine System Disorders
4	**CASP3**, **CCNA2**, **CDK4**, Ces, **DFFA**, **DNMT3A**, E2F1, **EIF4E**, **FLI1**, Hnrnpa1, KIF20A, **KLF5**, **MAPK7**, **MAPK12**, MCM6, **MDM2**, ME2, MPG, **MYC**, NR2E1, **PLK1**, PPARA, **PRC1**, SRM, **TOP2A**, TOP2B, YY1	26	16	Infectious Diseases, Organismal Development, Cellular Development
5	**ALOX5AP**, **APC**, ATG10, ATG2B, ATG4A, ATG4C, BMP1, CAPNS1, CTNNB1, **CYP2A6** (includes others), DIAPH3, **F11R**, FBLN2, FEN1, FSTL3, HIPK2, **IGFBP5**, **KRT7**, **MDFI**, MMP23B, PSAP, **RTKN**, **SMAD5**, **SPTB**, **TOM1**, **TP53**, TP63, **UNG**, USO1, USP9X, UVRAG, VAMP4, **VASN**, **VEGFA**, VMP1	22	14	Embryonic Development, Tissue Morphology, Cancer

IPA ingenuity pathway analysis was limited to experimentally validated mRNA targets of the sperm mircoRNAs with altered abundance due to a father’s HFD. Molecules that are in bold/underlined font are experimentally validated targets of the sperm microRNAs with altered abundance due to a HFD.

## Discussion

The 13 sperm borne miRNAs differentially regulated in a mouse model of paternal obesity may act as epigenetic signals transmitted to offspring, by targeting maternally stored mRNA transcripts in the fertilised egg, initiating a cascade of molecular events that reprogram embryonic development that ultimately impairs the long term metabolic and reproductive health of adult offspring. Furthermore, despite grand-offspring mice born to the male offspring displaying some similarities in metabolic and reproductive phenotypes to their offspring fathers, there was limited evidence that any of these microRNAs were also dysregulated in the offspring’s sperm. It must be noted that no dysregulation was observed in the sperm of the offspring presumably due to the variation of microRNA abundance detected within both groups of offspring. This suggests that either a different set of sperm borne microRNAs or other epigenetic/genetic marks are transmitted to grand-offspring that induce these phenotypes and that father to offspring signals may also comprise of more than sperm microRNAs. Taken together this implies that the set of sperm borne microRNAs identified as altered in these fathers’ sperm that were associated with programming in offspring, might be unique to the obese state, as male offspring born to obese fathers using this model of obesity were not themselves obese [19].

Of the four sperm microRNAs (mmu-miR-133b-3p, -196a-5p, 205-5p, 340-5p) that we previously reported as altered in fathers sperm due to this HFD regimen [19], miR-133b-3p was confirmed as up-regulated to a similar 3x fold increase. A further two (miR-205-5p, miR-340-5p) agreed with the direction of regulation due to a father’s HFD, but did not reach statistical significance due to the large variance detected across the limited sample size used in the Taqman array (*ie n* = 4), mainly between the HFD father samples. The last microRNA (miR-196a-5p) did not pass the quality test applied to all raw data prior to the LIMMA analysis, but interestingly it was detected in HFD fed fathers sperm but not in CD fed fathers (using a 35 Ct cut-off limit of detection), consistent with a large up-regulation as previously reported. This may be due to the multiplex nature of the method used for cDNA generation that might result in inconsistent representation of specific microRNAs in the cDNA versus a single assay in the subsequent qPCRs, or the variance of some microRNAs between the multiple cohorts. Thus highlighting the importance of qPCR confirmation of array data with a larger sample size from multiple cohorts.

It is possible that the sperm microRNAs that are differentially abundant in obese fathers enact their presumed perturbation to cellular functions by targeting maternally stored mRNAs post-fertilisation, as has recently been demonstrated for transmission of paternal stress cues [41]. Although, it must be noted that the microinjection of these microRNAs into early embryos would be required to provide direct causality of mRNA dysregulation, altered embryo development, and recapitulation of offspring phenotype. The top networks that the differentially abundant sperm borne microRNAs have been experimentally confirmed to modulate include functions such as cell death and survival, cellular development, cellular growth and proliferation, and embryonic development. Impairment to these functions might result in the delayed development, increased apoptosis, reduced cell numbers, and altered cell allocation that has been previously reported to occur in embryos fathered by males subjected to the same dietary regimen[52–54]. Part of the embryonic phenotype (of offspring) may well result from microRNA-145’s regulation of pluripotency, that has been demonstrated in human embryonic stem cells acting via repression of *OCT4*, *SOX2* and *KLF4* [55,56]. Moreover microRNA-143/145 have been demonstrated to be delivered by sperm, insofar as they were not detectable in oocytes but were present in both sperm and in early embryos soon after fertilisation [36]. Since sperm presumably deliver these microRNAs to the oocyte at fertilisation, their increased abundance in the sperm from obese fathers potentially leads to an increased inhibition of *OCT4*, *SOX2* and *KLF4*, reducing pluripotency within these embryos, which has important ramifications for the development of the pluripotent epiblast, which will ultimately develop into the fetal pole. A high fat diet has been shown to reduce the formation of the epiblast and also increases the amount of pregnancy loss in this mouse model of obesity [54]. Furthermore microRNA-135b (increased in obese males’ sperm) is a member of a family of microRNAs that target SIAH1, a protein essential to the first embryonic cell cleavage [57], and if its abundance is altered it has potential ramifications for the delayed cleavage observed for embryos fathered by males on the same HFD regimen [52–54]. Importantly, as these sperm microRNAs might potentially disrupt molecular pathways such as endocrine system disorders, tissue morphology, organismal injury & abnormalities, immune responses, and reproductive system disease this might initiate a post fertilisation molecular cascade that ultimately has a bearing on the subsequent health of offspring. This could possibly then partially explain the glucose homeostasis, adiposity, and reproductive defects evident in offspring fathered by males exposed to the same HFD regimen [19,20,22,58].

The differentially abundant sperm microRNAs may potentially also be acting upon DNA methylation post fertilisation. Indeed there are multiple lines of evidence that microRNAs can alter methylation patterns. Specifically microRNA-126-3p (increased in sperm from HFD fathers) binds the 3′-untranslated region of *DNMT1* mRNA, reducing its expression and leading to hypomethylation [59]. Interestingly, hypomethylation occurs in the germ cells of male mice exposed to the same HFD regimen [19]. MicroRNA-143 has been experimentally confirmed to represses *DNMT3A* expression in colon and breast cancer cell lines [60,61], and inhibit growth by causing apopotosis in leukaemia cells [62]. But whether micro-RNA-143-3p (increased in sperm from HFD fathers) is also capable of these functions remains to be determined. Furthermore microRNA-30a/microRNA-135b and microRNA-133b are also predicted to target *DNMT3A* and *DNMT3B*, respectively, for mRNA degradation. Overall the predicted targeting of components of the DNA methylation machinery provides the means for longer term changes to gene expression within the embryo and potentially even subsequent offspring. Furthermore given that offspring sperm does not overtly demonstrate the same compliment of differentially abundant microRNAs, DNA methylation might also be important for the phenotypic transmission from offspring to grand-offspring.

The tumour suppressor p53 is known to enhance post-transcriptional maturation of multiple miRNAs, including microRNA-143/microRNA-145 (increased in the sperm from HFD fathers), as a response to DNA damage [63]. Interestingly increased DNA damage has been previously reported to result from the model of male obesity used in this study [22,44,64] and this potentially triggers the increased abundance of p53 and thus microRNA-143/microRNA-145. This would likely occur in concert with the increase in the abundance of p53 due to diet induced obesity [65,66], possibly resulting from p53’s role in adipogenesis [67]. Moreover microRNA-143 is known to accelerate adipogenesis and is also increased in adipose tissue of mice fed a high fat diet [68]. Overall it remains possible that p53 acts upstream as a result of DNA damage and HFD consumption that ultimately leads to increased microRNA-143/microRNA-145 in sperm in HFD fathers.

MicroRNAs-145a-5p, 30a-5p, 126-3p, 184-3p, and 143-3p are the most differentially abundant as a result of diet induced obesity, perhaps indicating their potential to enact aberrant maternal mRNA decay in the embryo. Furthermore 8/13 microRNAs altered by a father’s obesity are amongst the microRNAs whose abundance are most altered in sperm during epididymal transit (mmu-miR- 126-3p, 126-5p, 135b-5p, 143-3p, 145a-5p, 30a-5p, 141-5p, and 184-3p [69]). Thus it remains a distinct possibility that the abundance of these microRNAs might be differentially acquired in obesity during epididymal transit, as the epididymis is not as protected from environmental insults as the testis. This altered microRNA transfer may well be enacted by epididymosomes [70] that may result from the effect of a father’s obesity, that then might also have altered microRNA content in epididymal epithelial cells, whose physiology is likely altered by systemic impacts of obesity [71].

Similar to our findings, the abundance of sperm borne microRNA-30a-5p was increased by a model of paternal stress [72]. This model of paternal stress resulted in behavioural and metabolic disturbances in offspring [41,72], similar to the metabolic disturbances reported for offspring sired by HFD fathers [19,20]. Interestingly HFD feeding in murine fathers tends to increase corticosterone [73], implying a degree of systemic stress is endured by males as a result of exposure to a HFD, which may be a common system to increase microRNA-30a-5p in sperm. Furthermore individual housing may also induce a degree of stress in mice, which might also contribute to this alteration. Thus it is possible that behavioural defects might also exist in offspring sired by HFD fathers, as observed in humans as an increased risk of autism spectrum disorders in children born to obese fathers [16,17]. Alternatively the model of paternal stress used might also cause metabolic derangements in the fathers themselves, which is then transmitted to offspring as previously reported, as stress is a known trigger for metabolic disturbances [74]. Although it has been previously demonstrated that the microinjection of a single microRNA can both alter the molecular makeup of the embryo and induce phenotypic outcomes in adult offspring (*eg* microRNA-221/222 [38]; microRNA-1 [37]; microRNA-124 [39]; microRNA-193-5p (partial phenotype) [41]). It has also been demonstrated that the co-ordinate regulation of molecular pathways by multiple microRNAs [41], entire sperm sncRNA extract [42], or specific sub-classes of sncRNA fractions (*ie* the tsRNA fraction [43]) are capable of recapitulating a complex phenotype. Whether microinjection of the amounts of microRNA, or other sncRNA fractions, actually present in a single sperm can also induce these molecular and phenotypic changes, remains to be determined.

The array used to interrogate sperm microRNAs was limited to 641 murine microRNAs of an estimated 1,400 that are present [75] and did not include investigation of the broader gamut of other small non-coding RNA. Regardless it must be noted that the same array was used to detect the 9 microRNAs altered by paternal stress that when microinjected into an early embryo were sufficient to recapitulate the offspring phenotype [41,72].

Diet and/or exercise interventions have been used in a similar model of diet induced obesity in fathers to demonstrate that this type of intervention strategy can be effective for ameliorating detrimental effects of paternal diet-induced obesity on metabolic parameters and sperm function [73], embryonic development [53], reproductive function of offspring [58], and metabolic health of offspring [20]. Moreover these interventions that mitigate the obesity in fathers occur concomitantly with a partial restoration of sperm borne microRNA content similar to that of control animals [20]. If the entirety of these findings are translatable to human cohorts sperm borne microRNAs could be potential readily assayable biomarkers of successful obesity interventions, to be used by obese men prior to conception to minimise detrimental health outcomes in his children.

We have demonstrated that the abundance of 13 sperm borne microRNAs are modulated by a father’s consumption of a high fat diet and speculate that the transmission of this altered microRNA content to the embryo at fertilisation acts to alter the mRNA content of the embryo, as has been previously demonstrated. This altered embryonic mRNA content then acts to alter growth trajectory previously observed for embryos fertilised by obese fathers and ultimately confers sub-optimal adult metabolic and reproductive outcomes described for offspring sired by obese fathers. Although the same microRNAs are most likely not altered in the sperm of male offspring, despite the presence of metabolic/reproductive phenotypes in grand-offspring sired by the male offspring. Thus we have identified a potential mechanism that may, in part, form the basis of obesity initiated paternal programming of the first generation of offspring.

## Supporting Information

S1 Table The degree of differential expression in a HFD father’s sperm of 371 mouse quality detectors that were ranked using LIMMA in R. (XLSX)

S2 Table Sperm microRNA expression in an expanded group from 3 cohorts of CD or HFD fed fathers (CD *n* = 13; HFD *n* = 14) as determined by qPCR. (DOCX)

S3 Table Mean Cts for 53 mouse sperm microRNAs that were only detected in either in CD or HFD fed father's sperm. (XLSX)

S4 Table The 311 experimentally validated mRNA targets of the 13 sperm microRNAs with differential abundance due to a father’s HFD and the microRNAs which target them. (XLSX)

## References

[1] Ng M, Fleming T, Robinson M, Thomson B, Graetz N, Margono C, et al Global, regional, and national prevalence of overweight and obesity in children and adults during 1980–2013: a systematic analysis for the Global Burden of Disease Study 2013. Lancet. 2014; 384: 766–781. 10.1016/S0140-6736(14)60460-8 24880830 PMC4624264

[2] Nguyen DM, El-Serag HB. The epidemiology of obesity. Gastroenterol Clin North Am. 2010; 39: 1–7. 10.1016/j.gtc.2009.12.014 20202574 PMC2833287

[3] Katzmarzyk PT, Ardern CI. Overweight and obesity mortality trends in Canada, 1985–2000. Can J Public Health. 2004; 95: 16–20. 14768735 10.1007/BF03403627 PMC6975909

[4] Chiang PH, Chang TY, Chen JD. Synergistic effect of fatty liver and smoking on metabolic syndrome. World J Gastroenterol. 2009; 15: 5334–5339. 10.3748/wjg.15.5334 19908343 PMC2776862

[5] Rato L, Alves MG, Cavaco JE, Oliveira PF. High-energy diets: a threat for male fertility? Obes Rev. 2014; 15: 996–1007. 10.1111/obr.12226 25346452

[6] Tamer Erel C, Senturk LM. The impact of body mass index on assisted reproduction. Curr Opin Obstet Gynecol. 2009; 21: 228–235. 10.1097/GCO.0b013e32832aee96 19395966

[7] Shayeb AG, Harrild K, Mathers E, Bhattacharya S. An exploration of the association between male body mass index and semen quality. Reprod Biomed Online. 2011; 23: 717–723. 10.1016/j.rbmo.2011.07.018 22019618

[8] Tunc O, Bakos HW, Tremellen K. Impact of body mass index on seminal oxidative stress. Andrologia. 2011; 43: 121–128. 10.1111/j.1439-0272.2009.01032.x 21382066

[9] Bakos HW, Henshaw RC, Mitchell M, Lane M. Paternal body mass index is associated with decreased blastocyst development and reduced live birth rates following assisted reproductive technology. Fertil Steril. 2011; 95: 1700–1704. 10.1016/j.fertnstert.2010.11.044 21145051

[10] Bygren LO, Kaati G, Edvinsson S. Longevity determined by paternal ancestors' nutrition during their slow growth period. Acta Biotheor. 2001; 49: 53–59. 11368478 10.1023/a:1010241825519

[11] Kaati G, Bygren LO, Edvinsson S. Cardiovascular and diabetes mortality determined by nutrition during parents' and grandparents' slow growth period. Eur J Hum Genet. 2002; 10: 682–688. 10.1038/sj.ejhg.5200859 12404098

[12] Danielzik S, Langnase K, Mast M, Spethmann C, Muller MJ. Impact of parental BMI on the manifestation of overweight 5–7 year old children. Eur J Nutr. 2002; 41: 132–138. 10.1007/s00394-002-0367-1 12111051

[13] Freeman E, Fletcher R, Collins CE, Morgan PJ, Burrows T, Callister R. Preventing and treating childhood obesity: time to target fathers. Int J Obes (Lond). 2012; 36: 12–15. 22005717 10.1038/ijo.2011.198

[14] Li L, Law C, Lo Conte R, Power C. Intergenerational influences on childhood body mass index: the effect of parental body mass index trajectories. Am J Clin Nutr. 2009; 89: 551–557. 10.3945/ajcn.2008.26759 19106237

[15] Whitaker KL, Jarvis MJ, Beeken RJ, Boniface D, Wardle J. Comparing maternal and paternal intergenerational transmission of obesity risk in a large population-based sample. Am J Clin Nutr. 2010; 91: 1560–1567. 10.3945/ajcn.2009.28838 20375189

[16] Murphy SK. Obesity: Paternal obesity—a risk factor for autism? Nat Rev Endocrinol. 2014; 10: 389–390. 10.1038/nrendo.2014.81 24889901

[17] Suren P, Gunnes N, Roth C, Bresnahan M, Hornig M, Hirtz D, et al Parental obesity and risk of autism spectrum disorder. Pediatrics. 2014; 133: e1128–1138. 10.1542/peds.2013-3664 24709932 PMC4006442

[18] Wahlqvist ML, Huang LY, Lee MS, Chiang PH, Chang YH, Tsao AP. Dietary quality of elders and children is interdependent in Taiwanese communities: a NAHSIT mapping study. Ecol Food Nutr. 2014; 53: 81–97. 10.1080/03670244.2013.772512 24437545

[19] Fullston T, Ohlsson Teague EM, Palmer NO, DeBlasio MJ, Mitchell M, Corbett M, et al Paternal obesity initiates metabolic disturbances in two generations of mice with incomplete penetrance to the F2 generation and alters the transcriptional profile of testis and sperm microRNA content. FASEB J. 2013; 27: 4226–4243. 10.1096/fj.12-224048 23845863

[20] McPherson NO, Owens JA, Fullston T, Lane M. Preconception diet or exercise interventions in obese fathers normalizes sperm microRNA profile and metabolic syndrome in female offspring. Am J Physiol Endocrinol Metab. 2015: ajpendo 00013 02015. 10.1152/ajpendo.00013.2015 25690453

[21] Ng SF, Lin RC, Laybutt DR, Barres R, Owens JA, Morris MJ. Chronic high-fat diet in fathers programs beta-cell dysfunction in female rat offspring. Nature. 2010; 467: 963–966. 10.1038/nature09491 20962845

[22] Fullston T, Palmer NO, Owens JA, Mitchell M, Bakos HW, Lane M. Diet-induced paternal obesity in the absence of diabetes diminishes the reproductive health of two subsequent generations of mice. Hum Reprod. 2012; 27: 1391–1400. 10.1093/humrep/des030 22357767

[23] Bohacek J, Mansuy IM. Molecular insights into transgenerational non-genetic inheritance of acquired behaviours. Nat Rev Genet. 2015; 16: 641–652. 10.1038/nrg3964 26416311

[24] Holland ML, Rakyan VK. Transgenerational inheritance of non-genetically determined phenotypes. Biochem Soc Trans. 2013; 41: 769–776. 10.1042/BST20130043 23697936

[25] Nilsson EE, Skinner MK. Environmentally induced epigenetic transgenerational inheritance of disease susceptibility. Transl Res. 2015; 165: 12–17. 10.1016/j.trsl.2014.02.003 24657180 PMC4148471

[26] Dadoune JP. Spermatozoal RNAs: what about their functions? Microsc Res Tech. 2009; 72: 536–551. 10.1002/jemt.20697 19283828

[27] Li Y, Wang HY, Wan FC, Liu FJ, Liu J, Zhang N, et al Deep sequencing analysis of small non-coding RNAs reveals the diversity of microRNAs and piRNAs in the human epididymis. Gene. 2012; 497: 330–335. 10.1016/j.gene.2012.01.038 22313525

[28] Ostermeier GC, Goodrich RJ, Moldenhauer JS, Diamond MP, Krawetz SA. A suite of novel human spermatozoal RNAs. J Androl. 2005; 26: 70–74. 15611569

[29] Ostermeier GC, Miller D, Huntriss JD, Diamond MP, Krawetz SA. Reproductive biology: delivering spermatozoan RNA to the oocyte. Nature. 2004; 429: 154 10.1038/429154a 15141202

[30] Bartel DP, Chen CZ. Micromanagers of gene expression: the potentially widespread influence of metazoan microRNAs. Nat Rev Genet. 2004; 5: 396–400. 10.1038/nrg1328 15143321

[31] Dong H, Lei J, Ding L, Wen Y, Ju H, Zhang X. MicroRNA: function, detection, and bioanalysis. Chem Rev. 2013; 113: 6207–6233. 10.1021/cr300362f 23697835

[32] Fabian MR, Sonenberg N, Filipowicz W. Regulation of mRNA translation and stability by microRNAs. Annu Rev Biochem. 2010; 79: 351–379. 10.1146/annurev-biochem-060308-103103 20533884

[33] Guo L, Lu Z. The fate of miRNA* strand through evolutionary analysis: implication for degradation as merely carrier strand or potential regulatory molecule? PLoS One. 2010; 5: e11387 10.1371/journal.pone.0011387 20613982 PMC2894941

[34] Sato F, Tsuchiya S, Meltzer SJ, Shimizu K. MicroRNAs and epigenetics. FEBS J. 2011; 278: 1598–1609. 10.1111/j.1742-4658.2011.08089.x 21395977

[35] Wang Y, Medvid R, Melton C, Jaenisch R, Blelloch R. DGCR8 is essential for microRNA biogenesis and silencing of embryonic stem cell self-renewal. Nat Genet. 2007; 39: 380–385. 10.1038/ng1969 17259983 PMC3008549

[36] Liu WM, Pang RT, Chiu PC, Wong BP, Lao K, Lee KF, et al Sperm-borne microRNA-34c is required for the first cleavage division in mouse. Proc Natl Acad Sci U S A. 2012; 109: 490–494. 10.1073/pnas.1110368109 22203953 PMC3258645

[37] Wagner KD, Wagner N, Ghanbarian H, Grandjean V, Gounon P, Cuzin F, et al RNA induction and inheritance of epigenetic cardiac hypertrophy in the mouse. Dev Cell. 2008; 14: 962–969. 10.1016/j.devcel.2008.03.009 18539123

[38] Rassoulzadegan M, Grandjean V, Gounon P, Vincent S, Gillot I, Cuzin F. RNA-mediated non-mendelian inheritance of an epigenetic change in the mouse. Nature. 2006; 441: 469–474. 10.1038/nature04674 16724059

[39] Grandjean V, Gounon P, Wagner N, Martin L, Wagner KD, Bernex F, et al The miR-124-Sox9 paramutation: RNA-mediated epigenetic control of embryonic and adult growth. Development. 2009; 136: 3647–3655. 10.1242/dev.041061 19820183

[40] Grandjean V, Fourre S, De Abreu DA, Derieppe MA, Remy JJ, Rassoulzadegan M. RNA-mediated paternal heredity of diet-induced obesity and metabolic disorders. Sci Rep. 2015; 5: 18193 10.1038/srep18193 26658372 PMC4677355

[41] Rodgers AB, Morgan CP, Leu NA, Bale TL. Transgenerational epigenetic programming via sperm microRNA recapitulates effects of paternal stress. Proc Natl Acad Sci U S A. 2015. 10.1073/pnas.1508347112 PMC4640733 26483456

[42] Gapp K, Soldado-Magraner S, Alvarez-Sanchez M, Bohacek J, Vernaz G, Shu H, et al Early life stress in fathers improves behavioural flexibility in their offspring. Nat Commun. 2014; 5: 5466 10.1038/ncomms6466 25405779

[43] Chen Q, Yan M, Cao Z, Li X, Zhang Y, Shi J, et al Sperm tsRNAs contribute to intergenerational inheritance of an acquired metabolic disorder. Science. 2016; 351: 397–400. 10.1126/science.aad7977 26721680

[44] Bakos HW, Mitchell M, Setchell BP, Lane M. The effect of paternal diet-induced obesity on sperm function and fertilization in a mouse model. Int J Androl. 2011; 34: 402–410. 10.1111/j.1365-2605.2010.01092.x 20649934

[45] WHO, Organisation WH. WHO laboratory manual for the examination and processing of human semen; ed.Wilson J.S., editor. Cambridge: Cambridge University Press 2010

[46] Mar JC, Kimura Y, Schroder K, Irvine KM, Hayashizaki Y, Suzuki H, et al Data-driven normalization strategies for high-throughput quantitative RT-PCR. BMC Bioinformatics. 2009; 10: 110 10.1186/1471-2105-10-110 19374774 PMC2680405

[47] Palmer NO, Fullston T, Mitchell M, Setchell BP, Lane M. SIRT6 in mouse spermatogenesis is modulated by diet-induced obesity. Reprod Fertil Dev. 2011; 23: 929–939. 10.1071/RD10326 21871212

[48] Anway MD, Cupp AS, Uzumcu M, Skinner MK. Epigenetic transgenerational actions of endocrine disruptors and male fertility. Science. 2005; 308: 1466–1469. 10.1126/science.1108190 15933200 PMC11423801

[49] Franklin TB, Russig H, Weiss IC, Graff J, Linder N, Michalon A, et al Epigenetic transmission of the impact of early stress across generations. Biol Psychiatry. 2010; 68: 408–415. 10.1016/j.biopsych.2010.05.036 20673872

[50] Dias BG, Ressler KJ. Parental olfactory experience influences behavior and neural structure in subsequent generations. Nat Neurosci. 2014; 17: 89–96. 10.1038/nn.3594 24292232 PMC3923835

[51] Carone BR, Fauquier L, Habib N, Shea JM, Hart CE, Li R, et al Paternally induced transgenerational environmental reprogramming of metabolic gene expression in mammals. Cell. 2010; 143: 1084–1096. 10.1016/j.cell.2010.12.008 21183072 PMC3039484

[52] Binder NK, Hannan NJ, Gardner DK. Paternal diet-induced obesity retards early mouse embryo development, mitochondrial activity and pregnancy health. PLoS One. 2012; 7: e52304 10.1371/journal.pone.0052304 23300638 PMC3531483

[53] McPherson NO, Bakos HW, Owens JA, Setchell BP, Lane M. Improving metabolic health in obese male mice via diet and exercise restores embryo development and fetal growth. PLoS One. 2013; 8: e71459 10.1371/journal.pone.0071459 23977045 PMC3747240

[54] Mitchell M, Bakos HW, Lane M. Paternal diet-induced obesity impairs embryo development and implantation in the mouse. Fertil Steril. 2011; 95: 1349–1353. 10.1016/j.fertnstert.2010.09.038 21047633

[55] Foygel K, Choi B, Jun S, Leong DE, Lee A, Wong CC, et al A novel and critical role for Oct4 as a regulator of the maternal-embryonic transition. PLoS One. 2008; 3: e4109 10.1371/journal.pone.0004109 19129941 PMC2614881

[56] Xu N, Papagiannakopoulos T, Pan G, Thomson JA, Kosik KS. MicroRNA-145 regulates OCT4, SOX2, and KLF4 and represses pluripotency in human embryonic stem cells. Cell. 2009; 137: 647–658. 10.1016/j.cell.2009.02.038 19409607

[57] Pang RT, Liu WM, Leung CO, Ye TM, Kwan PC, Lee KF, et al miR-135A Regulates Preimplantation Embryo Development through Down-Regulation of E3 Ubiquitin Ligase Seven in Absentia Homolog 1A (SIAH1A) Expression. PLoS One. 2011; 6: e27878 10.1371/journal.pone.0027878 22132158 PMC3222661

[58] McPherson NO, Fullston T, Bakos HW, Setchell BP, Lane M. Obese father's metabolic state, adiposity, and reproductive capacity indicate son's reproductive health. Fertil Steril. 2014; 101: 865–873 e861. 10.1016/j.fertnstert.2013.12.007 24424359

[59] Zhao S, Wang Y, Liang Y, Zhao M, Long H, Ding S, et al MicroRNA-126 regulates DNA methylation in CD4+ T cells and contributes to systemic lupus erythematosus by targeting DNA methyltransferase 1. Arthritis Rheum. 2011; 63: 1376–1386. 10.1002/art.30196 21538319

[60] Ng EK, Li R, Shin VY, Siu JM, Ma ES, Kwong A. MicroRNA-143 is downregulated in breast cancer and regulates DNA methyltransferases 3A in breast cancer cells. Tumour Biol. 2014; 35: 2591–2598. 10.1007/s13277-013-1341-7 24218337

[61] Ng EK, Tsang WP, Ng SS, Jin HC, Yu J, Li JJ, et al MicroRNA-143 targets DNA methyltransferases 3A in colorectal cancer. Br J Cancer. 2009; 101: 699–706. 10.1038/sj.bjc.6605195 19638978 PMC2736825

[62] Shen JZ, Zhang YY, Fu HY, Wu DS, Zhou HR. Overexpression of microRNA-143 inhibits growth and induces apoptosis in human leukemia cells. Oncol Rep. 2014; 31: 2035–2042. 10.3892/or.2014.3078 24626955

[63] Suzuki HI, Yamagata K, Sugimoto K, Iwamoto T, Kato S, Miyazono K. Modulation of microRNA processing by p53. Nature. 2009; 460: 529–533. 10.1038/nature08199 19626115

[64] McPherson NOB, H. W., Setchell BP, Owens JA, Lane M. Improving metabolic health in obese male mice via diet and exercise restores embryo development and fetal growth. PLOS One. 2013; 8: e71459 10.1371/journal.pone.0071459 23977045 PMC3747240

[65] Bogazzi F, Raggi F, Russo D, Bohlooly YM, Sardella C, Urbani C, et al Growth hormone is necessary for the p53-mediated, obesity-induced insulin resistance in male C57BL/6J x CBA mice. Endocrinology. 2013; 154: 4226–4236. 10.1210/en.2013-1220 23913444

[66] Yahagi N, Shimano H, Matsuzaka T, Najima Y, Sekiya M, Nakagawa Y, et al p53 Activation in adipocytes of obese mice. J Biol Chem. 2003; 278: 25395–25400. 10.1074/jbc.M302364200 12734185

[67] Huang Q, Liu M, Du X, Zhang R, Xue Y, Zhang Y, et al Role of p53 in preadipocyte differentiation. Cell Biol Int. 2014; 38: 1384–1393. 10.1002/cbin.10334 25045020

[68] Takanabe R, Ono K, Abe Y, Takaya T, Horie T, Wada H, et al Up-regulated expression of microRNA-143 in association with obesity in adipose tissue of mice fed high-fat diet. Biochem Biophys Res Commun. 2008; 376: 728–732. 10.1016/j.bbrc.2008.09.050 18809385

[69] Nixon B, Stanger SJ, Mihalas BP, Reilly JN, Anderson AL, Tyagi S, et al The MicroRNA Signature of Mouse Spermatozoa Is Substantially Modified During Epididymal Maturation. Biol Reprod. 2015; 93: 91 10.1095/biolreprod.115.132209 26333995

[70] Belleannee C, Calvo E, Caballero J, Sullivan R. Epididymosomes convey different repertoires of microRNAs throughout the bovine epididymis. Biol Reprod. 2013; 89: 30 10.1095/biolreprod.113.110486 23803555

[71] Nixon B, Stanger SJ, Mihalas BP, Reilly JN, Anderson AL, Dun MD, et al Next Generation Sequencing Analysis Reveals Segmental Patterns of microRNA Expression in Mouse Epididymal Epithelial Cells. PLoS One. 2015; 10: e0135605 10.1371/journal.pone.0135605 26270822 PMC4535982

[72] Rodgers AB, Morgan CP, Bronson SL, Revello S, Bale TL. Paternal stress exposure alters sperm microRNA content and reprograms offspring HPA stress axis regulation. J Neurosci. 2013; 33: 9003–9012. 10.1523/JNEUROSCI.0914-13.2013 23699511 PMC3712504

[73] Palmer NO, Bakos HW, Owens JA, Setchell BP, Lane M. Diet and exercise in an obese mouse fed a high-fat diet improve metabolic health and reverse perturbed sperm function. Am J Physiol Endocrinol Metab. 2012; 302: E768–780. 10.1152/ajpendo.00401.2011 22252945

[74] Tamashiro KL, Sakai RR, Shively CA, Karatsoreos IN, Reagan LP. Chronic stress, metabolism, and metabolic syndrome. Stress. 2011; 14: 468–474. 10.3109/10253890.2011.606341 21848434

[75] Kawano M, Kawaji H, Grandjean V, Kiani J, Rassoulzadegan M. Novel small noncoding RNAs in mouse spermatozoa, zygotes and early embryos. PLoS One. 2012; 7: e44542 10.1371/journal.pone.0044542 22984523 PMC3440372

