# Supplementary material for: Sperm microRNA Content Is Altered in a Mouse Model of Male Obesity, but the Same Suite of microRNAs Are Not Altered in Offspring’s Sperm
Source: PLoS One. 2016 Nov 4;11(11):e0166076. doi: 10.1371/journal.pone.0166076 (PMC5096664; doi:10.1371/journal.pone.0166076)
Supplement: S2 Table — (DOCX) [file pone.0166076.s002.docx]

**Supplementary Table S2. Sperm microRNA expression in an expanded group from 3 cohorts of CD or HFD fed fathers (CD *n*=13; HFD *n* =14) as determined by qPCR.**

| **Mature miRNA Up-regulated in HFD** | **Taqman Assay ID** | **CD Mean (ΔCt)** | **HFD Mean (ΔCt)** | **HFD FC (ΔΔCt)** | ***p* value** |
| --- | --- | --- | --- | --- | --- |
| mmu-miR-337-3p | 002532 | 10.95±0.29 | 10.02±0.40 | 3.15 | 0.0001 |
| mmu-miR-126-3p | 002228 | 0.30±0.32 | 0.09±0.31 | 3.26 | 0.0002 |
| mmu-miR-126-5p | 000451 | 4.01±0.28 | 3.90±0.26 | 2.37 | 0.0010 |
| mmu-miR-135b-5p | 002261 | 6.20±0.17 | 4.93±0.16 | 2.60 | 0.0010 |
| mmu-miR-133b-3p | 002247 | 6.78±0.24 | 5.23±0.23 | 3.38 | 0.0041 |
| mmu-miR-143-3p | 002249 | 0.11±0.25 | -1.46±0.24 | 4.06 | 0.0080 |
| mmu-miR-145a-5p | 002278 | -2.17±0.30 | -3.53±0.29 | 3.41 | 0.0086 |
| mmu-miR-376a-3p | 001069 | 9.79±0.35 | 10.07±0.34 | 2.45 | 0.0158 |
| mmu-miR-30a-5p | 000417 | -0.74±0.20 | -0.66±0.20 | 1.63 | 0.0197 |
| mmu-miR-141-5p | 002513 | 8.57±0.41 | 7.13±0.46 | 2.72 | 0.0372 |
| mmu-miR-136-5p | 002511 | 6.77±0.25 | 7.20±0.24 | 1.84 | 0.0454 |
| mmu-miR-669n | 197143_mat | 8.91±0.29 | 8.16±0.29 | 1.89 | NS |
| mmu-miR-412-5p | 464537_mat | 12.57±0.50 | 12.10±0.48 | 1.54 | NS |
| mmu-miR-669c | 464620_mat | 9.88±0.38 | 8.77±0.35 | 1.48 | NS |
| mmu-miR-1969 | 121131_mat | 10.13±0.25 | 9.62±0.27 | 1.43 | NS |
| mmu-miR-376b | 002451 | 10.19±0.17 | 10.12±0.28 | 1.23 | NS |
| mmu-miR-669d-5p | 002808 | 8.22±0.26 | 7.94±0.29 | 1.22 | NS |
| mmu-miR-466b-3p | 464896_mat | 7.37±0.55 | 7.39±0.29 | 1.11 | NS |
| mmu-miR-669l-5p | 121149_mat | 8.67±0.16 | 8.67±0.17 | 1.04 | NS |
| mmu-miR-879-3p | 002473 | 11.55±0.20 | 11.55±0.25 | 1.00 | NS |
| **Down-regulated in HFD** |  |  |  |  |  |
| mmu-miR-184-3p | 000485 | 0.36±0.21 | 0.16±0.20 | -1.52 | 0.0090 |
| mmu-miR-1961 | 197391_mat | -3.18±0.27 | -2.29±0.28 | -1.86 | 0.0414 |
| mmu-miR-139-3p | 002546 | 8.96±0.32 | 9.45±0.48 | -1.40 | NS |
| mmu-miR-672-5p | 002327 | 3.66±0.20 | 3.50±0.19 | -1.21 | NS |
| mmu-miR-466a-3p | 002586 | 6.93±0.26 | 7.15±0.26 | -1.15 | NS |
| mmu-miR-467h | 002809 | 8.76±0.23 | 8.85±0.31 | -1.06 | NS |
| mmu-miR-92a-3p | 000430 | 0.39±0.15 | -0.01±0.14 | -1.04 | NS |
| mmu-miR-150-5p | 000473 | -0.40±0.22 | -1.21±0.22 | -1.01 | NS |
| **Endogenous controls** |  |  |  |  |  |
| mmu-miR-10a-5p | 000387 | -0.35±0.05 | -0.36±0.05 | 1.09 | NS |
| mmu-miR-195a-5p | 000494 | -0.36±0.05 | -0.35±0.05 | 1.08 | NS |
| U6 snRNA | 001973 | 7.04±0.26 | 7.47±0.24 | 1.48 | NS |

All data is presented as Mean ± SEM. Mean dCt are derived from the geometric mean of mmu-miR-10a-5p and mmu-miR-195-5p (the least variable microRNAs across all samples in the present experimental setup). *p* value is derived from univariate general linear modelling. microRNA annotations from Sanger miRBase, release 19 (August 2012).

CD control diet, HFD high fat diet, FC fold change (of HFD vs CD by ΔΔCt method), NS not significant between CD/HFD groups (*p*>0.05).
